# Natural and artificial abiotic factors: impacts on the seminal quality of Dorper rams in a semiarid region

**DOI:** 10.1007/s00484-026-03138-z

**Published:** 2026-02-24

**Authors:** Gabriel Thales Barboza Marinho, Héliton Pandorfi, Marcos Vinícius da Silva, Weslley Amaro da Silva, Raquel Desenzi, André Mariano Batista, Gledson Luiz Pontes de Almeida, Cristiane Guiselini, Alex Souza Moraes

**Affiliations:** 1 Department of Agricultural Engineering, Federal Rural University of Pernambuco– UFRPE, Dom Manoel de Medeiros avenue, SN, Dois Irmãos, Recife, Pernambuco 52171-900 Brazil; 2 Center for Agricultural and Environmental Sciences, Federal University of Maranhão, BR 222 km 4, s/n, Boa Vista, Chapadinha, Maranhão 65500000 Brazil; 3 Department of Veterinary Medicine, Federal Rural University of Pernambuco, Dom Manoel de Medeiros avenue, SN, Dois Irmãos, Recife, Pernambuco 52171-900 Brazil; 4 Department of Chemestry, Federal Rural University of Pernambuco, Dom Manoel de Medeiros avenue, SN, Dois Irmãos, Recife, Pernambuco 52171-900 Brazil

**Keywords:** Climatization, Heat stress, Principal components, Seminal quality, Thermal comfort

## Abstract

The climatic conditions of northeastern Brazil can induce heat stress, reducing the weight, production, and reproductive rates of sheep. This study aimed to analyze the dynamics of abiotic factors, comfort indices on the seminal quality of Dorper sheep under normal local meteorological conditions and adiabatic evaporative cooling. The study involved 20 rams aged 18 ± 2 months, weighing an average of 70 ± 3 kg, raised in a semiarid region of Brazil from April to June 2023. The animals were kept in an open pen for 22 days and for 43 days under the influence of an adiabatic evaporative cooling system (AECS). Air temperature (AT, ºC), relative humidity (RH, %), and black globe temperature (BGT, ºC) were monitored every 15 min daily, with semen collected and analyzed every three weeks, totaling four collections. Thermal characterization of the pen was determined by temperature and humidity indices, black globe temperature and humidity, and specific enthalpy (kJ kg-1). Semen characteristics were analyzed using a completely randomized design with two treatments and 20 replicates, with means compared using the t-test (*p* ≤ 0.05). Meteorological variables and comfort indices underwent descriptive statistical analysis. Principal Component Analysis (PCA) was applied to these variables and seminal characteristics. During the driest periods, AECS increased pen RH by 8% and reduced AT by 1.24 °C, maintaining THI values below critical levels. The PCA revealed that only sperm plasma membrane integrity (PMI) significantly responded to environmental changes, increasing by 16% during AECS operation, allowing a regression model with R² greater than 0.72 for estimating PMI.

## Introduction

The Northeast region of Brazil, characterized by an arid and semi-arid geographical domain, stands out for its predominance in goat and sheep farming, constituting a fundamental activity that provides demographic stability and profitability for the local population (Silva et al. 2016), with the state of Pernambuco having the second largest sheep herd in the northeast (IBGE, 2022). However, although the sheep herd is growing, productivity remains low (Santos et al. 2023).

Pernambuco’s semiarid region experiences high temperatures almost all year round, with average annual minimum and maximum temperatures of 24 °C and 29 °C, respectively (INMET 2020). During the warmest hours, the daily maximum can reach between 35 and 38 °C, which can lead animals in this region to heat stress, one of the notable limitations to animal production in the tropics (Rashamol et al. 2018).

Heat stress is the result of a combination of meteorological elements, including wind speed, air temperature, relative humidity and high solar radiation, which negatively affect thermal comfort and productivity (Thornton et al. 2022). This phenomenon occurs when the characteristics of the environment are outside the limit of the animal’s thermoneutral zone, which according to Baêta and Souza (2010) is between 25 and 30 °C for sheep, leading them to activate mechanisms to overcome the adversities of the environment, affecting food consumption, weight gain and reproductive rates (Thornton et al. 2022).

To unify the effects of meteorological elements, physiological and behavioral indicators are commonly used in judging animal thermal comfort or stress (Silva et al., 2019a, b, Marins et al. 2020; Borges et al. 2021). Good examples of indicators used in the thermal characterization of animal rearing environments are the temperature and humidity index (THI), proposed by Thom (1959) and adapted by Silva et al. (2020), the black globe temperature and humidity index (BGTHI), proposed by Buffington et al. (1981) and the specific enthalpy, proposed by Rodrigues et al. (2011).

Experiments indicate adverse effects of weather variables on spermatogenesis and the quality of animal semen (Freitas et al. 2020; Shahat et al. 2020; Capela et al. 2022; Lyrio et al. 2023; Shakeel and Yoon 2023), where in rams, Hedia et al. (2020) observed that seminal quality can decrease during the spring and summer months, with their reproduction strongly influenced by the environment and the heritability of their sperm characteristics ranging from low to moderate (Serrano et al. 2021).

Rahimi et al. (2021) and Marinho et al. (2023) indicate that current global climate conditions are propitious to the occurrence of heat stress, showing a significantly increasing trend when compared to future climate change scenarios. In an attempt to overcome this scenario, small ruminants have several adaptive mechanisms such as behavioral modifications and physiological, biochemical and molecular changes (Spandan et al. 2022).

However, although heat stress is known to impair male fertility, most evidence comes from studies using artificially induced stress, such as climatic chambers or scrotal heating (Maurya et al. 2016; Kalyan De et al., 2017; Shahat et al. 2021). Consequently, little is known about how natural climatic fluctuations in semiarid regions affect seminal characteristics of rams under real production conditions. Furthermore, studies evaluating whether microclimate improvement can influence seminal quality in small ruminants remain limited, representing an important knowledge gap for sheep production in tropical and semiarid environments.

To avoid animals having to activate their adaptive mechanisms, it is necessary to use techniques to adjust the environment to the animals’ conditions. In this regard, evaporative cooling has emerged as a simple and economical solution, reducing the dry bulb temperature of the air by adding humidity (Foroushani and Amon 2022). This process involves the exchange of energy resulting from the interaction between air and water molecules (Godyń et al. 2020), where the sensible heat transferred from the air to the water exclusively meets the energy demand required for evaporation, with the migration of thermal energy playing a significant role in lowering the air temperature (Simmons and Lott 1996).

Although adiabatic evaporative cooling systems (AECS) have been successfully used in different livestock production systems to mitigate heat load (Godyń et al. 2020; Raza et al. 2020; Santos et al., 2024), information on their effects on reproductive traits, particularly seminal quality in rams, remains limited. Given the high temperatures that characterize semiarid regions, where environmental conditions frequently exceed the thermoneutral zone for sheep (Joy et al. 2020; Leite et al. 2021; Marinho et al. 2023), evaluating AECS as an environmental management strategy may help support improvements in reproductive efficiency under field conditions.

In this context, the objective of this study was to evaluate the influence of natural climatic conditions (Phase 1) and an adiabatic evaporative cooling system (Phase 2) on the seminal quality of rams by integrating meteorological monitoring, thermal comfort indices, and multivariate analyses. By addressing this gap, the study provides evidence on whether microclimate improvement through AECS can modulate seminal traits under real semiarid production conditions, thereby strengthening the understanding of environmental factors that shape reproductive performance in dorper sheep.

## Materials and methods

### Characterization of the study area and selection of animals

The experiment was carried out from April to June 2023 (65 days) in a commercial sheep production unit located in the municipality of Garanhuns, Agreste mesoregion, Garanhuns microregion, state of Pernambuco (08°49’12’’S; 36°29’11’’O and altitude of 866 m). According to the Köppen classification, the climate in this region is As, tropical with a dry summer (Beck et al. 2018) with an average temperature of 20 °C and 891.4 mm of average annual rainfall (INMET 2020), distributed mainly between April and July. For the months of April, May and June, the climatological normals for maximum temperature are 26, 24 and 23 °C, respectively, based on the 1991–2020 climatological normals published by the Brazilian National Institute of Meteorology (INMET 2020).

### Installation and food management

**Fig. 1:**
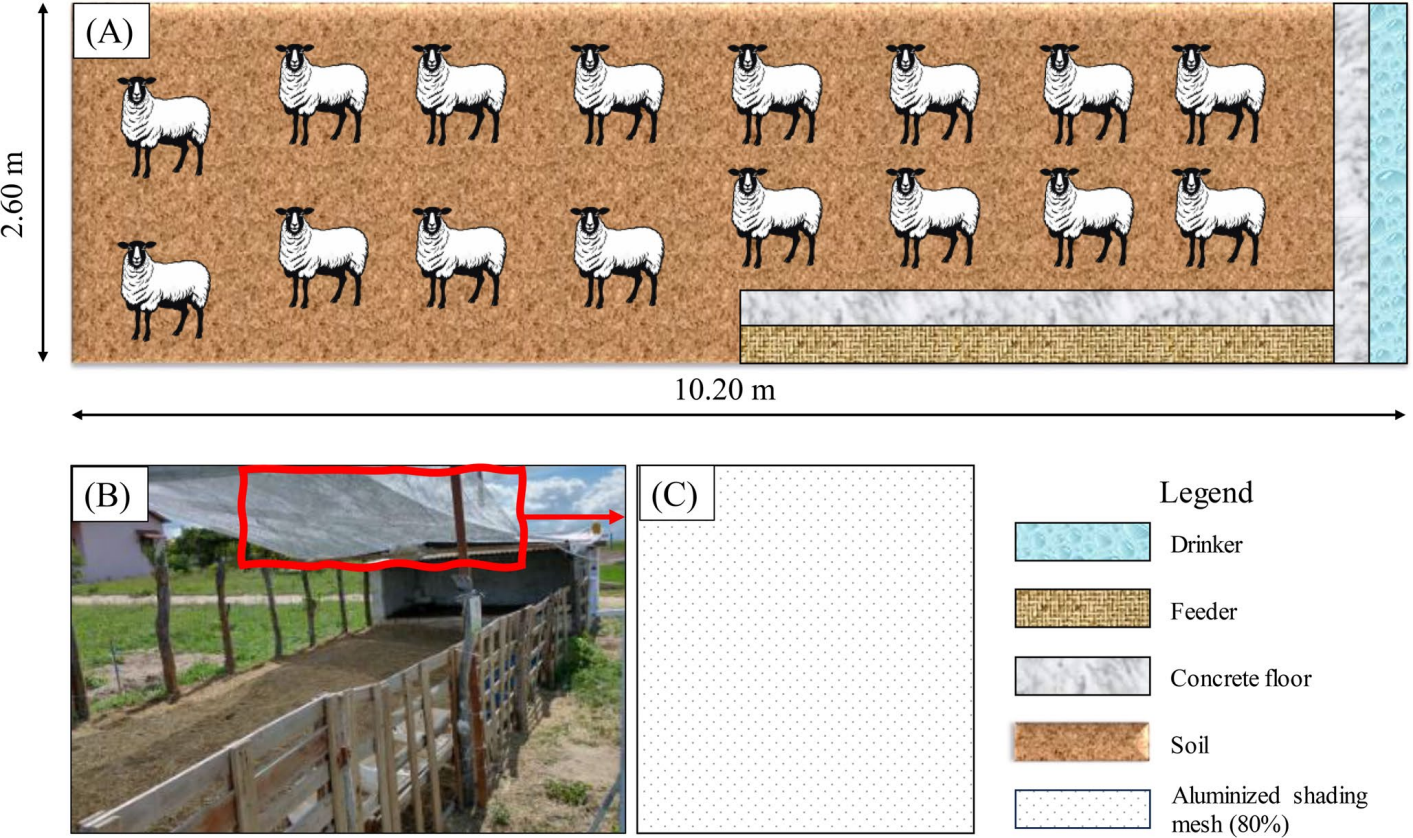
Illustration of the pen with the drinker and feeder (**A**); animal housing pen (**B**); aluminized shading mesh (80%) (**C**)

The 20 animals were housed in an open production pen measuring 2.60 × 10.20 m (Fig. 1), which had an aluminized shading mesh (80%), equipped with a drinker and feeder, most of which was made of exposed soil and a concrete floor only in the area where the drinker was located. Feeding was carried out twice a day directly at the feeder, based on roughage (*Panicum*) and concentrate (corn and soybean meal, 18–20% crude protein), with vitamins and minerals supplied through premix, a homogeneous mixture of micro-ingredients.

The animals were subjected to two different conditions: Phase 1 (Control), 22 days in a pen with normal local meteorological conditions, and Phase 2 (Treatment), 43 days of artificial air conditioning using an adiabatic evaporative cooling system (AECS), in which two evaporative air conditioners were activated. The mist was formed by the centrifugal effect of a central disk with an average flow rate of 7 L h⁻¹. Independent motors with a propeller speed of 1,750 rpm and a central disk speed of 3,450 rpm, with an average flow speed of 2.5 m s⁻¹. Forced ventilation was carried out using axial fans with a flow rate of 1,200 m³ h⁻¹ at 1,780 rpm and a propeller diameter of 11’’, providing an air flow at an average speed of 3.4 m s⁻¹. The system was operated daily from 8 a.m. to 5 p.m.

In Phase 1 (Control), two semen collections and analyses were carried out on the rams, spaced 21 days apart. This interval was chosen to minimize handling stress while still allowing changes in spermatogenesis to be reflected in subsequent ejaculates. In rams, a full spermatogenic process lasts approximately 49–57 days, whereas one cycle of the seminiferous epithelium lasts about 10–11 days (Cardoso and Queiroz 1988). Therefore, the 21-day interval used here corresponds to roughly two cycles of the seminiferous epithelium, permitting a significant proportion of spermatozoa in each collection to originate from different spermiogenesis cycles, although it does not represent a complete renewal of the spermatogenic population. After the second semen collection, the AECS was activated, initiating Phase 2 (Treatment), where new semen collections and analyses were carried out after 21 and 42 days of the system’s operation. Meteorological variables and the determination of thermal comfort indices were monitored daily during the two phases.

Although the use of a sequential design, with the same animals exposed first to natural conditions and subsequently to the AECS, can be considered a limitation compared to parallel groups evaluated simultaneously, this approach was chosen to reduce inter-individual variability in seminal parameters (Peris-Frau et al., 2025). Furthermore, the total experimental duration (65 days) covered the complete spermatogenic cycle in rams, ensuring that changes in semen quality parameters could be detected during the study period.

### Recording meteorological variables

A datalogger was installed inside the pens, in a meteorological shelter, at a height of 1.50 m from the floor. In order to compare the effectiveness of the AECS, a datalogger of the same model was also installed outside the pen, out of reach of the cooling system, in a meteorological shelter 1.50 m from the floor. The data was recorded in real time every 15 min throughout the study period. Because the monitoring took place at two different and consecutive times, the datalogger outside the pen also helped to characterize possible natural changes in temperature and humidity at the site of the experiment, making it possible to verify the true efficiency of the AECS at the site and period of the study.

The dataloggers used were the HOBO 4 model (Onset Computer Corporation Bourne, MA, USA) for recording air temperature (AT, °C), relative humidity (RH, %) and black globe temperature (BGT, °C). The datalogger’s measurement range for AT is −20 to 70 °C (± 0.35 °C) and for RH between 5 and 95% (± 2.5%). The BGT was measured using a hollow polyethylene sphere, painted matt black, 15 cm in diameter, into which a thermal sensor (thermistor) was inserted, recording the BGT and immediately storing it in the datalogger.

### Comfort indices

For thermal characterization of the environment during the study period, the temperature and humidity index (THI), proposed by Thom (1959), the black globe temperature and humidity index (BGTHI), proposed by Buffington et al. (1981) and the specific enthalpy (SE, kJ kg^− 1^), proposed by Rodrigues et al. (2011) were calculated using the following Eqs. 1, 2 and 3, respectively:

1 $$\:THI=AT+0.36DPT+41.5$$

 where, AT – is the air temperature (°C); DPT – is the dew point temperature (°C).

2 $$\:BGTHI=BGT+0.36DPT-330.08$$

 where, BGT – is the black globe temperature (K); DPT – is the dew point temperature (K).

3 $$\:SE=1.006AT+\frac{RH}{{P}_{atm}}{10}^{\frac{7.5AT}{237.3+AT}}.\left(71.28+0.052AT\right)$$

 where, AT – air temperature (^o^C); RH - relative humidity (%); P_atm_ - atmospheric pressure (mmHg).

### Semen collection and analysis

Semen was collected through an artificial vagina with the assistance of a female in estrus. Immediately after the procedure, the ejaculate volume was measured in a graduated glass tube, and the semen was taken for laboratory analysis. The semen samples were evaluated for total motility (0–100%) and progressive vigor (0–5, referring to the intensity of forward movement) using an optical microscope with 100X magnification (Batissaco et al. 2020; Van de Hoek et al. 2022). To do this, a drop of semen (1:400 dilution in Tris solution – 3.63 g Tris (hydroxymethyl) aminomethane, 1.99 g citric acid, and 0.5 g fructose in 100 ml of distilled water, pH 7.0) was placed on a slide and covered with a heated coverslip. For mass sperm motility (0–5, referring to the wave motion of the sperm mass), a drop of pure semen was placed on a slide and then observed under an optical microscope at 40X magnification (Van de Hoek et al. 2022). Sperm concentration (×10⁹/ml) was calculated using a Neubauer chamber (1:400 dilution in distilled water) (Almadaly et al. 2016). Plasma membrane integrity was analyzed using the eosin-nigrosin stain, where a smear was prepared from a drop of semen (1:400 dilution in distilled water) and 10 µl of eosin-nigrosin (Carvajal-Serna et al. 2022). The smears were observed under an optical microscope (400X), where 200 cells per sample were counted and classified as intact (unstained) or non-intact (stained) membranes (Carvajal-Serna et al. 2022).

### Statistical analysis

#### Experimental design

The semen characteristics data from Phase 1 (Control) and Phase 2 (Treatment) were compared using a **t-test** (*p* ≤ 0.05). The experimental design adopted was entirely randomized, with two treatments and 20 replicates, characterized by the number of animals available per treatment. According to the effects model described in Eq. 4:

4 $$\:y_{ij}=\mu\:+{\tau\:}_i+\varepsilon_{ij}$$

 where, $\:{y}_{ij}$ - is the observed value for the response variable obtained for the i-th treatment in its j-th repetition; $\:\mu\:$ - is the average of the means of each treatment; $\:{\tau\:}_{i}$ - is the effect of treatment i on the observed value $\:{y}_{ij}$; $\:\varepsilon_{ij}$ - is the experimental error associated with the observed value $\:{y}_{ij}$.

The meteorological variables and comfort indices were subjected to descriptive statistical analysis to obtain the mean, median, minimum, maximum, standard deviation (SD), coefficients of variation (CV, %), asymmetry and kurtosis. The percentage value of the CV was categorized as low (CV < 12%), medium (CV = 12–24%), or high (CV > 24%) according to the criteria of Warrick and Nielsen (1980).

#### Principal component analysis

Principal Component Analysis (PCA) was carried out in order to assess possible correlations between seminal quality variables, meteorological variables and comfort indices, associated with climate control factors. The Kaiser criterion was applied, considering only eigenvalues greater than 1, which result in components containing a significant amount of information from the original data (Kaiser 1958). The PCA comprised three distinct phases: generation, selection and interpretation of the components analyzed. After determining the optimum number of principal components, the contribution of each variable to the most relevant components was analyzed, culminating in the Principal Components Matrix (PCM).

#### Regression analysis

Multiple linear regression models were determined based on the results of the PCA. These models were based on the variables with the highest correlation with the Principal Components (PCs), assuming the greatest representativeness of the data set. The regression model was based on Eq. 5 below:

5 $$\:\mathrm{Y}={\upbeta\:}0+{\upbeta\:}1\mathrm{x}1+{\upbeta\:}2\mathrm{x}2+{\upbeta\:}3\mathrm{x}3+\:{\upbeta\:}4\mathrm{x}4+\:{\upepsilon\:}$$

 where, Y - response variable (semen quality parameters); x1, x2, x3 e x4 - predictor variables (meteorological variables and comfort indices); β0, β1, β2, β3 and β4 - regression coefficients; ε - experimental error.

## Results and discussion

### Meteorological data and comfort indices

Table 1 shows the descriptive statistics for black globe temperature in the external environment (BGT (EE) and in the pen (BGT (PEN), air temperature in the external environment (AT (EE) and in the pen (AT (PEN), relative humidity in the external environment (RH (EE) and in the pen (RH (PEN), temperature and humidity index in the external environment (THI (EE) and in the pen (THI (PEN), black globe temperature and humidity index in the external environment (BGTHI (EE) and in the pen (BGTHI (PEN) and specific enthalpy in the external environment (SE (EE) and in the pen (SE (PEN). The mean and median values were similar to each other for all variables except relative humidity, which is a good indication that the data shows or is close to a normal distribution (Silva et al. 2021; Oliveira-Júnior et al. 2022; Marinho et al. 2023).

**Table 1:** Summary of descriptive statistics for meteorological data in phase 1 and 2

Phase 1								
	Mean	Median	SD	CV	K	A	Min	Max
BGT (EE)	29.14	29.29	3.00	10.30	−0.49	−0.09	21.91	36.69
BGT (PEN)	28.27	28.53	2.78	9.83	−0.36	−0.19	21.75	34.92
AT (EE)	27.74	27.91	2.70	9.73	−0.55	−0.05	21.66	34.68
AT (PEN)	27.64	27.87	2.63	9.52	−0.38	−0.14	21.68	34.03
RH (EE)	59.11	57.42	13.16	22.26	−0.53	0.42	30.86	93.73
RH (PEN)	60.08	58.13	13.06	21.74	−0.23	0.51	31.83	98.09
THI (EE)	76.15	76.21	2.49	3.27	−0.52	−0.01	70.50	82.54
THI (PEN)	75.99	76.19	2.48	3.27	−0.34	−0.04	70.25	82.30
BGTHI (EE)	77.46	77.45	2.78	3.60	−0.45	−0.04	70.66	84.69
BGTHI (PEN)	76.53	76.65	2.65	3.46	−0.32	−0.08	70.07	83.52
SE (EE)	61.65	61.68	3.07	4.97	0.23	−0.11	50.85	70.55
SE (PEN)	61.95	61.82	3.41	5.51	−0.10	0.07	51.33	72.77
Phase 2								
	Mean	Median	SD	CV	K	A	Min	Max
BGT (EE)	27.21	27.20	2.73	10.02	−0.52	0.13	20.80	36.75
BGT (PEN)	25.99	25.74	2.67	10.27	−0.23	0.37	19.88	38.66
AT (EE)	26.48	26.51	2.45	9.25	−0.55	0.12	20.74	35.01
AT (PEN)	25.24	25.15	2.38	9.44	−0.01	0.24	19.69	36.05
RH (EE)	68.61	68.32	11.58	16.87	−0.92	−0.01	37.50	92.27
RH (PEN)	73.45	73.93	9.95	13.55	−0.81	−0.15	45.17	96.13
THI (EE)	75.18	75.27	2.39	3.18	−0.46	0.03	68.90	83.37
THI (PEN)	73.73	73.68	2.31	3.13	−0.10	0.15	67.58	84.17
BGTHI (EE)	75.82	75.89	2.66	3.51	−0.42	0.07	68.87	85.02
BGTHI (PEN)	74.38	74.18	2.60	3.50	−0.33	0.32	67.65	86.67
SE (EE)	63.27	63.06	3.12	4.93	0.41	0.25	53.60	74.06
SE (PEN)	61.98	61.96	3.95	6.38	0.47	0.17	50.00	78.68

*SD* Standard deviation, *CV* Coefficient of variation, *K* Kurtosis, *A* Asymmetry, *Min* Minimum, *Max* Maximum.

**Fig. 2:**
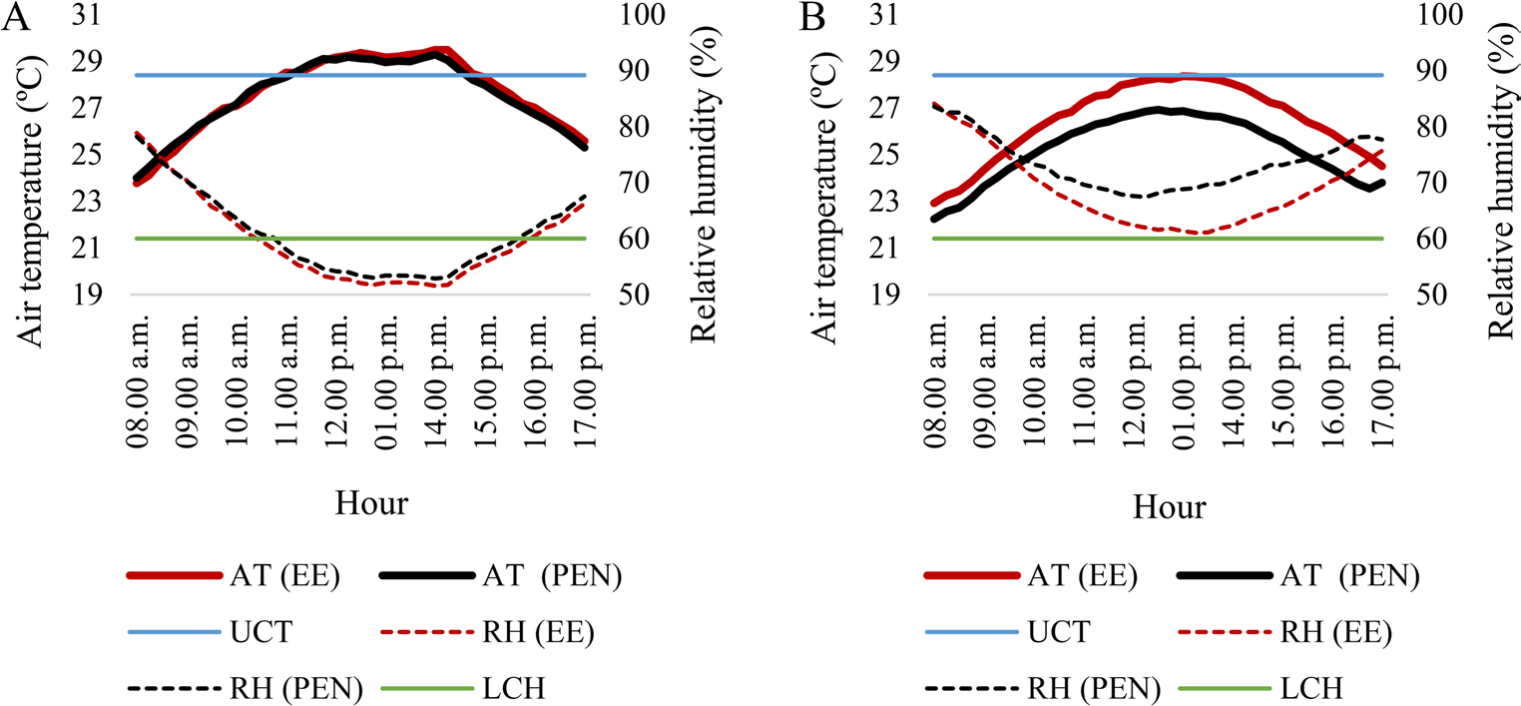
Average hourly values of air temperature and relative humidity in Phase 1 (**A**) and Phase 2 (**B**). AT (EE) – Air temperature in the external environment; AT (PEN) – Air temperature in the pen; RH (EE) – Relative humidity in the external environment; RH (PEN) – Relative humidity in the pen; UCT – Upper Critical Temperature; LCH – Lower Critical Humidity

The CV indicated high data homogeneity, being considered low (< 12%) for all the variables monitored, with the exception of relative humidity (RH), which had coefficients of variation considered as medium (Warrick and Nielsen 1980). This variability can be explained by the fact that the average RH in the region quickly dropped from 80% at 8 a.m. to values below 55% in the driest time interval (11 a.m. to 3 p.m.), a condition below the lower critical humidity (LCH) of 60% for sheep farming (Lees et al. 2019), rising again at 4 p.m. (Fig. 2A). There was a decrease in CV values in Phase 2 (Table 1), especially in the values observed in the pen, indicating that the AECS mitigated the drop in RH values at the driest times (Fig. 2B), where an average increase of 7.12% was observed in the relative humidity of the pen compared to the external environment.

The SD values were higher in Phase 1 for all variables, indicating less variability in the data during Phase 2 (Table 1). The kurtosis values were negative for all variables, indicating a less steep distribution curve than normal (Bono et al. 2019), regardless of phase or environment. In Phase 1, all variables except RH exhibited a negative asymmetry close to zero, suggesting a slightly above-average concentration of the data (Hatem et al. 2022). In Phase 2, the trend changed (Table 1), with only RH showing positive asymmetry values, indicating an above-average concentration for this variable and below-average concentration for the others.

In order to identify which regions of the state of Pernambuco would be most suitable for different pure breeds of sheep, McManus et al. (2014) studied the critical limits for environmental control variables to assess the distribution of sheep flocks in Brazil and indicated an upper critical temperature (UCT) of 28.4 °C for Dorper sheep. Marinho et al. (2023), who carried out a bioclimatic zoning study in the state of Pernambuco, seeking to characterize the main climatic zones suitable for the production of different breeds of sheep, based on non-parametric statistics and kriging maps of the temperature and humidity index (THI), corroborate the zones pointed out by McManus et al. (2014).

Daily mean air temperature (AT) and relative humidity (RH) in the pen differed significantly between the two phases. Using Welch’s t-test, AT decreased from 27.64 ± 2.63 °C in Phase 1 to 25.24 ± 2.38 °C in Phase 2 (t = 4.59; df = 37.86; *p* < 0.001), while RH increased from 60.08 ± 13.06% to 73.45 ± 9.95% (t = − 5.39; df = 35.18; *p* < 0.001). These findings statistically support the differences illustrated in Fig. 2, confirming that the environmental conditions during Phase 2 were consistently cooler and more humid than those observed in Phase 1.

In Fig. 2A, it can be seen that during Phase 1, at the hottest times, the average air temperature exceeded the TCS value (McManus et al. 2014), with an average value of 27.74 and 27.64 °C for the period in the external environment and in the pen (Table 1), respectively. During Phase 2, the air temperature did not exceed the UCT value (Fig. 2B), with the pen having an average temperature of 25.24 °C (Table 1), 1.24 °C lower than the external environment. Almeida Neto et al. (2014) investigated the impact of different AECS exposure periods in the waiting barn of Girolando cows during the winter season in semiarid Pernambuco, analyzing comfort indices, physiological variables and milk production, and observed a reduction in temperature of 1.4 to 2.7 °C and an increase in relative humidity of 7 to 13%, where evaporation was affected by the climatic configuration of the winter season, determined by low temperatures and high relative humidity.

The BGT was higher in the external environment in both phases (Table 1), showing that the shading mesh in the pen worked properly, blocking part of the radiant energy. In Phase 2, the values were even lower due to the lower air temperature and higher relative humidity during this period. Corroborating the findings of this study, Silva et al., 2019a, b), using meteorological data from northeastern Brazil in order to develop equations for predicting BGT, found a significant positive correlation (*p* < 0.0001) of BGT with solar radiation and air temperature, and a significant negative correlation of this variable with relative humidity (*p* < 0.0001), observing no significant effects of wind speed on BGT.

Daily mean THI differed significantly between the two phases (Table 1). According to Welch’s t-test, THI decreased from 75.99 ± 2.48 in Phase 1 to 73.73 ± 2.31 in Phase 2 (t = 4.56; df = 37.73; *p* < 0.001), confirming that the thermal load experienced by the rams was consistently lower during Phase 2.

**Fig. 3:**
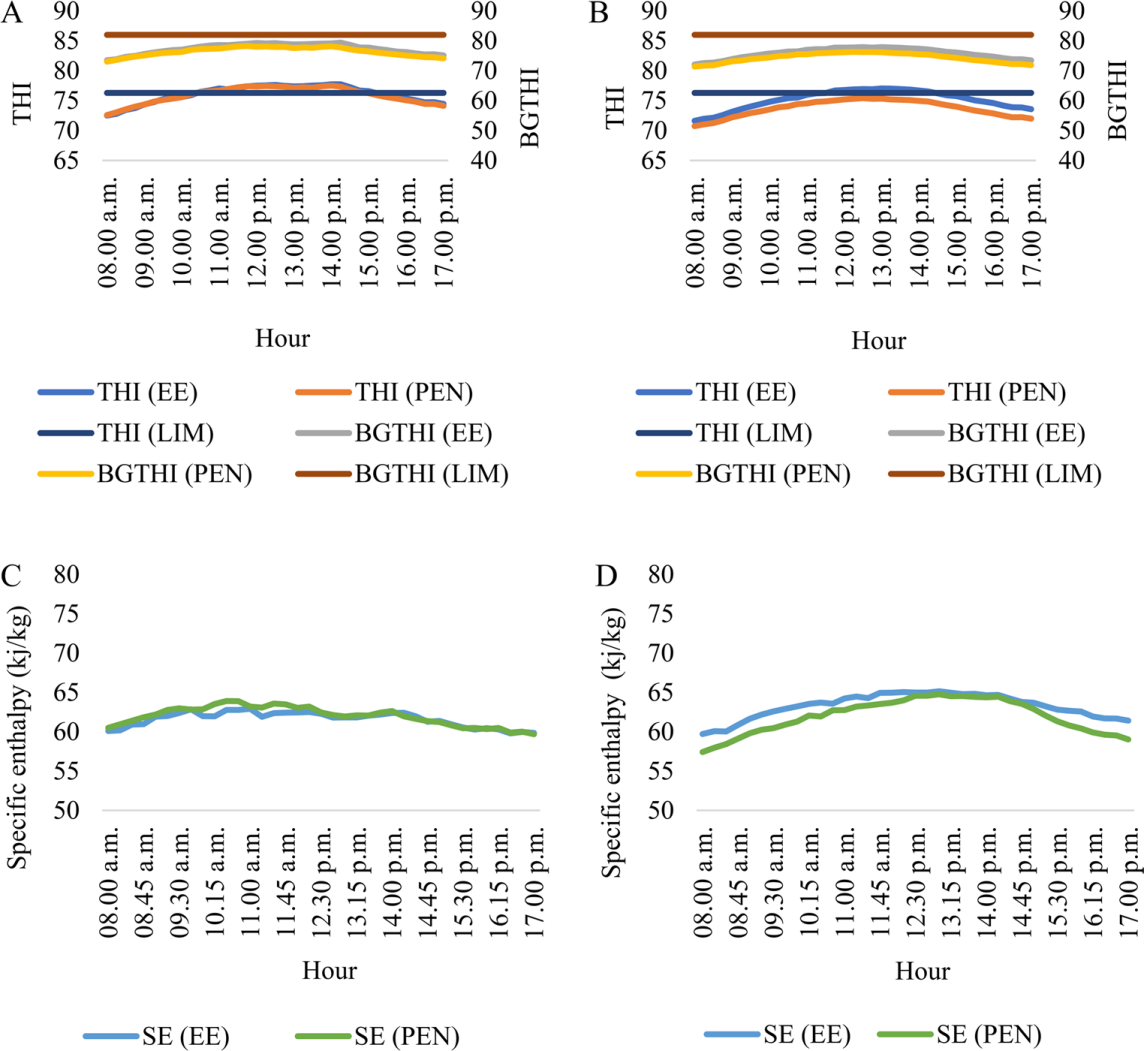
Monitoring of average hourly THI and BGTHI values in Phase 1 (**A**) and Phase 2 (**B**) and specific enthalpy in Phase 1 (**C**) and Phase 2 (**D**). THI (LIM) - THI limit value for rams; BGTHI (LIM) - BGTHI limit value for rams

Neves et al. (2009), estimating the THI limit value based on the respiratory rate of white sheep in Agreste Pernambucano, reached a value of 76.3. Throughout Phase 1, it was observed that the pen presented an unfavorable situation, exceeding this limit value during the interval of 10.30 am to 14.15 pm. During Phase 2, there was a reduction in the THI values in the pen compared to the external environment, with ideal conditions throughout the day. From 11.45 a.m. to 2.15 p.m. the THI values in the external environment exceeded the critical value of 76.3 (Fig. 3B). According to Table 1, the AECS promoted an average reduction of 1.92% in THI during the hours it was in operation. Bleizgys et al. (2023) analyzed the operation of the AECS inside a cattle barn in a hot desert climate with an average air temperature and relative humidity of 21.51 °C and 70.74%, respectively, and found that THI values inside the barn were around 1.6% lower than in the external area.

Daily mean BGTHI values also differed significantly between phases. Using Welch’s t-test, BGTHI decreased from 76.53 ± 2.65 in Phase 1 to 74.38 ± 2.60 in Phase 2 (t = 3.97; df = 39.66; *p* < 0.001). Throughout the experiment, BGTHI values were lower than 82 (Table 1), characterizing the absence of heat stress for sheep (Neves et al. 2009; Mascarenhas et al. 2023). The highest values were observed during Phase 1, with means of 77.46 and 76.53, in the external environment and in the pen, respectively (Table 1). During Phase 2, the average hourly BGTHI in the pen remained below 76.30 (Fig. 3B), with the AECS providing a 1.89% reduction in BGTHI compared to the external environment, which in turn showed values close to 78 during the most critical periods.

Abreu et al. (2016), evaluating the spatial variation of BGTHI in open facilities (3.20 × 4.00 m) with and without AECS, where the average temperature and relative humidity on site during the experiment period was 26.55 °C and 60.65%, respectively, observed an average amplitude of the index of only 2.1.

In contrast to THI and BGTHI, daily mean SE values did not differ significantly between phases. Welch’s t-test indicated means of 61.95 ± 3.41 in Phase 1 and 61.98 ± 3.95 in Phase 2 (t = 0.07; df = 41.33; *p* = 0.94), demonstrating that enthalpy remained stable throughout the experimental period.

Regardless of the condition, the specific enthalpy values did not exceed 65 kJ kg-1 of dry air, characterizing values that are not usually associated with heat stress (Silveira et al. 2024). The AECS promoted a 2.04% reduction in specific enthalpy between the external environment and the pen (Table 1), however, it was observed that during the hours of greatest variation in air temperature and humidity between one environment and the other, the difference in specific enthalpy was minimal (Fig. 3D). This is due to the dominant influence of air humidity in the calculation of this physical parameter, which makes it possible to measure the energy present in the air parcel, keeping the specific enthalpy constant (Kapilan et al., 2023). Its influence may be related to the body’s difficulty in releasing energy to the environment through evaporation, however, in situations of moderate temperatures, the exclusive impact of air humidity does not constitute a state of heat stress (Castro Júnior and Silva, 2021).

**Fig. 4:**
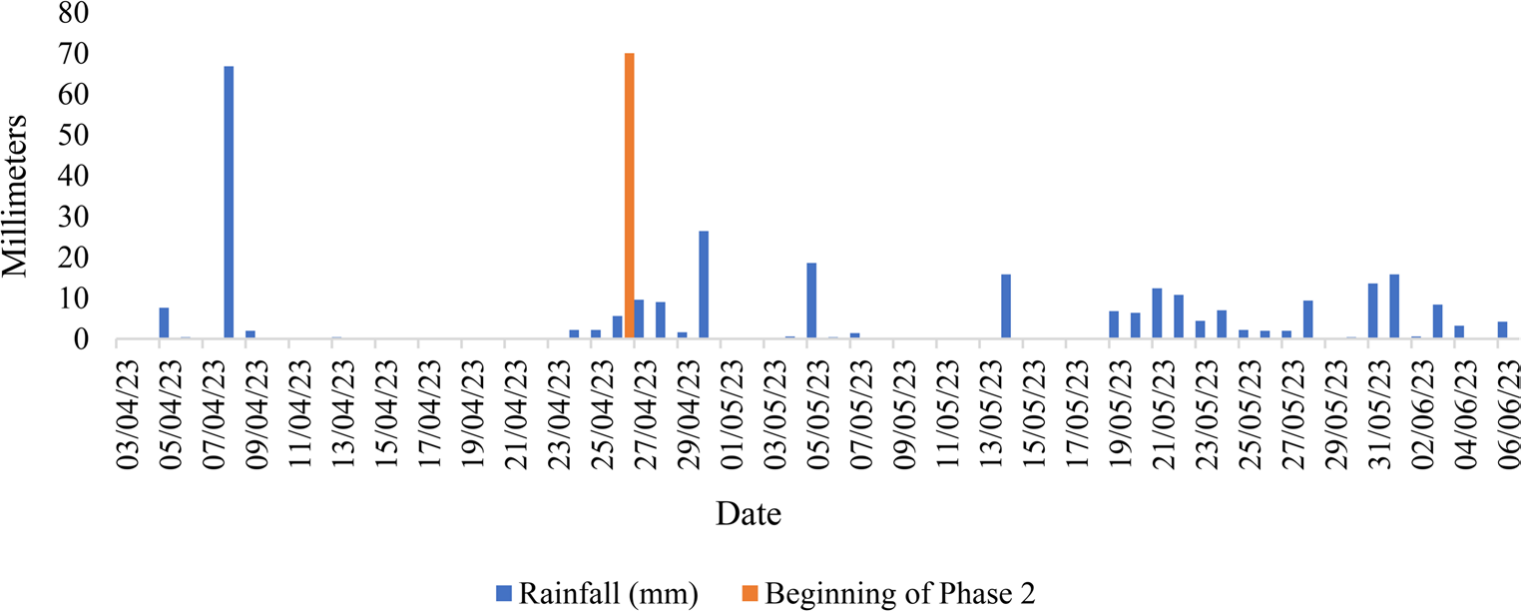
Daily rainfall accumulated during the study period

Following the variables in Table 1 in the external environment, it can be seen that in Phase 2 there was a natural decrease in the variables BGT, AT, THI, BGTHI and SE, as well as an increase in RH. This phenomenon can be attributed to the increase in rainy days, which can be seen in Fig. 4, constructed from National Meteorological Institute data (INMET 2024), after the activation of the AECS, resulting in an increase in the average relative humidity and an intrinsic decrease in air temperature, exerting a negative influence on the efficiency of the AECS (Kashif et al. 2017; Raza et al. 2020).

Although these climatic shifts were described above, it is important to recognize them as a limitation of the study, since the natural increase in humidity during the rainy period may have influenced the magnitude of the AECS response. Future research should evaluate the system under different seasonal conditions.

### Semen quality parameters

**Fig. 5:**
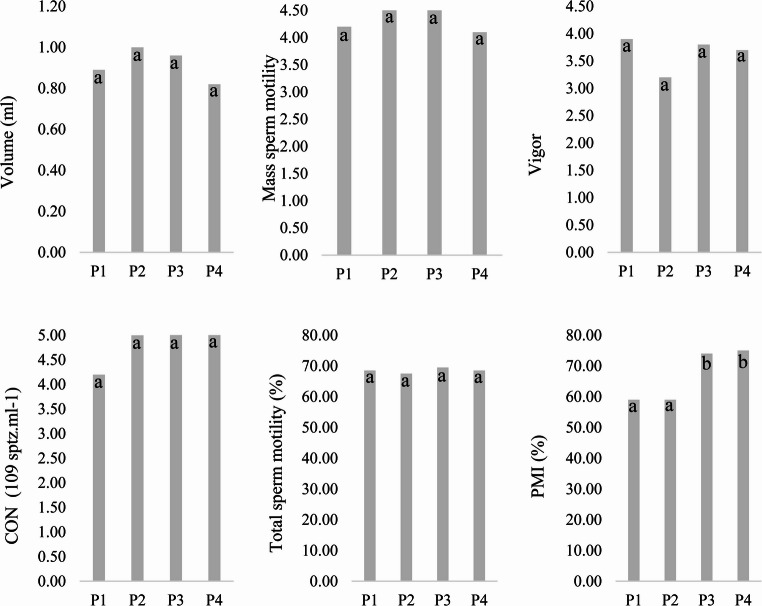
Seminal parameters before and after air conditioning with AECS. P1–21 fisrt days of natural climatic conditions; P2–21 last days of natural climatic conditions; P3–21 fisrt days of artificial climatization; P4–21 last days of artificial climatization; sptz - Spermatozoa; PMI - Plasma membrane integrity; CON – Sperm concentration. Means with different superscripts differ significantly (*p* ≤ 0.05)

The average seminal volume showed no significant differences among the four periods evaluated (*p* > 0.05), ranging from 0.82 ± 0.40 ml (P4) to 1.00 ± 0.51 ml (P2). All values were close to the expected average of approximately 1 ml for rams, according to CBRA (2013). Sperm concentration also did not differ significantly across periods (*p* > 0.05), although numerical variation was observed, with means ranging from 4.20 ± 1.64 × 10⁹ sptz/ml (P1) to 5.04 ± 1.95 × 10⁹ sptz/ml (P4). These concentrations were consistently higher than the reference range of 1–3 × 10⁹ sptz/ml established by CBRA (2013), indicating good testicular efficiency throughout the experiment (Fig. 5).

Mass sperm motility also remained statistically unchanged among the four periods (*p* > 0.05). The values ranged from 4.10 ± 0.99 (P4) to 4.90 ± 0.32 (P3), all of which are considered satisfactory for sheep semen. Despite numerical oscillation between natural and artificially cooled phases, the differences were not significant.

Sperm vigor showed a similar pattern, with no significant variation among phases (*p* > 0.05), oscillating between 3.20 ± 1.69 (P2) and 3.90 ± 1.64 (P1). All values were above the reference score of 3 recommended by CBRA (2013), indicating adequate sperm progressiveness throughout the study.

Among all seminal variables, only plasma membrane integrity (PMI) showed a statistically significant difference between phases (*p* ≤ 0.05). The mean PMI increased markedly from 59.0 ± 5.2% in P1 to 59.0 ± 4.1% in P2, and reached substantially higher values during the climatized phases—74.0 ± 4.4% (P3) and 75.0 ± 6.0% (P4). These findings indicate that the adiabatic evaporative cooling system had a positive effect on maintaining sperm membrane integrity during spermatogenesis.

This improvement aligns with previous reports showing that PMI is one of the seminal traits most sensitive to thermal stress, reflecting both the cell’s ability to maintain osmotic and structural stability and the efficiency of spermatogenesis under varying environmental conditions (Residiwati et al. 2020; Gloria et al. 2021; Sabés-Alsina et al. 2019). Unlike the other seminal parameters, PMI responded clearly to the environmental modulation provided by AECS, supporting the hypothesis that improved microclimatic conditions, particularly lower temperature and more stable humidity, favor membrane preservation.

Although many studies evaluate sperm quality after experimentally induced heat stress (e.g., scrotal insulation or controlled environmental chambers), few have assessed how natural variations in air temperature and humidity affect semen under real field conditions (van Wettere et al. 2021), and even fewer have attempted to improve seminal quality by modifying the rearing environment.

Although no significant differences were detected for total sperm motility, sperm concentration, and vigor, it should be noted that the sample size (20 rams evaluated at four time points) may have limited the statistical power to detect small treatment effects on these traits. Therefore, the non-significant results for these variables should be interpreted with caution, and future studies including larger numbers of animals and repeated ejaculates are recommended to confirm whether adiabatic evaporative cooling has more subtle impacts on these aspects of seminal quality.

While the natural climatic conditions during the experiment did not reach extreme values, the inferential analyses demonstrated that the differences between phases were statistically significant. Even moderate reductions in AT, RH, THI and BGTHI during the AECS period indicate that small but sustained changes in microclimate can reduce thermal load and improve at least one seminal trait (PMI).

### Principal component analysis

The principal components generated by multivariate analysis for the meteorological variables (air temperature, relative humidity and black globe temperature), comfort indices (THI, BGTHI and specific enthalpy) and seminal characteristics (volume, mass sperm motility, total sperm motility, vigor, concentration and plasma membrane integrity) of the rams during the study period are shown in Table 2. PC1 and PC2 had an eigenvalue greater than 1, according to the criteria established by Kaiser (1958), with eigenvalues of 6.51 and 1.87, respectively. Components 1 and 2 have an accumulated variance of 69.90%.

**Table 2:** Principal components of meteorological variables, comfort indices and seminal characteristics

Variable	PC1	PC2
AT	0.388	−0.062
RH	−0.389	−0.009
BGT	0.369	0.011
THI	0.386	−0.076
BGTHI	0.386	−0.076
SE	0.384	−0.064
VOL	0.032	0.093
MSM	0.005	−0.404
TSM	−0.063	−0.590
VIG	−0.034	−0.608
CON	−0.067	0.231
PMI	−0.326	−0.193
Eigenvalue	6.51	1.87
Proportion	54.30	15.60

*AT* Air temperature, *RH* Relative humidity, *BGT* Black globe temperature, *THI* Temperature and humidity index, *BGTHI* Black globe temperature and humidity index, *SE* Specific enthalpy, *TSM* Total Sperm Motility, *MSM* Mass Sperm Motility, *CON* Concentration, *VIG* Vigor, *VOL* Volume, *PMI* Plasma membrane integrity.

Leite et al. (2022) investigated the relationship between semen quality traits and oxidative stress in cattle and obtained accumulated variance of 73.4% and 74.8%. Sun et al. (2022), analyzed the effect of environmental temperature on semen quality and seminal plasma metabolites in buffalo and found an accumulated variance of 61.2 and 62.7%.

**Fig. 6:**
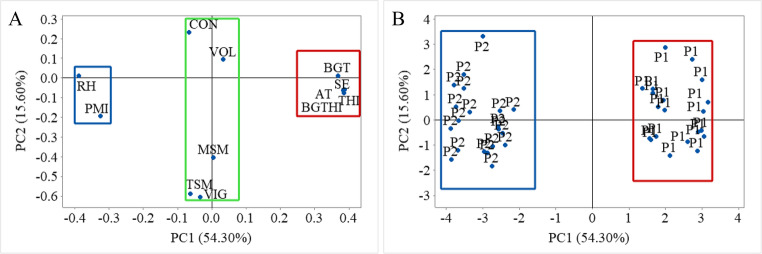
Principal components of meteorological variables, comfort indices, specific enthalpy and seminal characteristics (**A**); Principal components of animals in Phase 1 and Phase 2 (**B**). P1 - Animals in Phase 1; P2 - Animals in Phase 2

Analyzing Fig. 6, it can be seen that the inverse relationship between air temperature and relative humidity was best explained by Principal Component 1 (PC1), corroborating the results of Silva et al. (2023a) and Silva Silva et al. (2023b) who observed the same phenomenon when studying the association between abiotic, physiological and behavioral factors in pigs. Air temperature (AT), black globe temperature (BGT), temperature and humidity index (THI), black globe temperature and humidity index (BGTHI) and specific enthalpy (SE) are variables that showed a positive correlation with each other (Table 2), results that corroborate the findings of Silva et al. (2020),Silveira et al. (2021) and (Silva et al., 2023c), who, when studying the thermal comfort of cattle and goats in the Brazilian semiarid region, observed the same. It was possible to see the existence of three large groups (Fig. 6A), with the variables AT, BGT, THI and BGTHI, together with specific enthalpy, forming one of them. Looking at Fig. 6B, it can be seen that the animals in Phase 1 and Phase 2 were grouped at different extremes along the PC1 axis. From this cluster, it can be seen that the animals in Phase 1 were directly associated with the group formed by air temperature (Fig. 6A), while in Phase 2, this association was observed with relative humidity.

The seminal characteristics of concentration, volume, mass sperm motility, vigor and total sperm motility do not fit into either of the two groups formed by AT and RH, with positions on the graph close to the y-axis, suggesting that these parameters were not significantly influenced by the treatment carried out. Unfortunately, in the literature there is a lack of studies investigating the effect of the use of the adiabatic evaporative cooling system on improving the seminal quality of animals, with studies on this topic being focused on carrying out thermal insults, whether environmental or localized. Maurya et al. (2016), evaluating the effect of heat stress on Malpura rams conditioned in a climate chamber at 42 °C and 55% relative humidity for 6 h for 45 days, observed a significant decrease (*p* ≤ 0.05) in sperm volume, mass sperm motility and concentration. Seifi-Jamadi et al. (2020), testing the effect of seasons on Belgian Blue bulls, observed no effect on semen volume (*p* > 0.05) when the environmental temperature was increased from 35 °C to 8 °C, but an increase in sperm concentration was noted (*p* ≤ 0.05). Freitas et al. (2020) observed a negative effect of the combination of high temperatures and humidity on the vigor of sperm from girolando cattle. According to Rizzoto et al. (2020), mild, short-term heat stress can significantly affect sperm total motility 14 to 21 days after exposure, with a negative impact on the developmental stages of the spermatid and spermatocyte, where the formation of the flagellum occurs.

There was a negative correlation between plasma membrane integrity (PMI) and the group formed by air temperature, black globe temperature and comfort indices, corroborating the studies by Residiwati et al. (2020) and Gloria et al. (2021), who observed that the PMI of sperm from Belgian Blue cattle was significantly reduced under heat stress. The plasma membrane not only outlines the cell, being responsible for its integrity, but is also important for mitochondrial activity (Sabés-Alsina et al. 2019) and cellular interactions, such as the attachment of sperm to the oviduct epithelium or the penetration of the oocyte by the sperm cell, being directly related to semen quality (Akbarinejad et al. 2020). The significant variation in the PMI of sperm throughout the phases can be explained by the animal’s inability to lose heat properly or by the inefficient elimination of cells damaged by apoptosis (Durairajanayagam et al. 2015; Hamilton et al. 2016). Figure 6B shows that the animals in Phase 2 were directly related to the variables PMI and RH, which were positively correlated with each other, showing that the highest percentage of animals with intact plasma membranes occurred during this period, characterized by an increase in RH and a decrease in AT.

According to the principal component analysis (Fig. 6) it was possible to determine the main predictor variables, thus enabling the development of multiple regression models to characterize the seminal quality parameters (Table 3). The variables BGTHI and BGTHI were not taken into account in the modeling because they explain the same variance in the data as AT. Due to multicollinearity, a condition that occurs when some predictor variables in the model are highly correlated with other predictor variables, increasing the variance of the regression coefficients and making them unstable, it was not possible to obtain equations involving AT, BGT and specific enthalpy at the same time, as they provide redundant information. Therefore, based on the ease of obtaining the possible predictor variables, the following models were determined for each seminal quality parameter.

**Table 3:** Models for determining seminal quality parameters

Equation	*R*²	*p*-value
PMI = −351 + 9.38 AT + 2.738 RH – 0.564 BGT	0.729	< 0.001
VOL = 2.7 + 0.025 AT − 0.0190 RH − 0.0501 BGT	0.025	0.820
MSM = −6.9 + 0.597 AT + 0.024 RH − 0.254 BGT	0.147	0.122
TSM = −664 + 21.24 AT + 3.60 RH − 2.85 BGT	0.253	0.014
VIG = −36.2 + 0.790 AT + 0.2290 RH + 0.1494 BGT	0.169	0.079
CON = 28.9 − 0.37 AT − 0.132 RH − 0.229 BGT	0.041	0.672

*AT* Air temperature, *RH* Relative humidity, *TSM* Total Sperm Motility, *MSM* Mass Sperm Motility, *CON* Concentration, *VIG* Vigor, *VOL* Volume,* PMI* Plasma membrane integrity

It can be seen in Table 3 that only the model for plasma membrane integrity, a seminal quality characteristic that showed a high correlation with the variables monitored during the study period, presented a coefficient of determination considered to be strong and at the same time presented a p-value ≤ 0.05, representing a statistically significant association between the response variable and the predictor terms.

## Conclusions

The adiabatic evaporative cooling system was effective in maintaining the air temperature and relative humidity of the pen within the thermoneutral zone of Dorper sheep, and was also effective in preventing the THI values from reaching critical levels at times when the air temperature was highest.

The exploratory analysis of the data showed that the plasma membrane integrity of sperm from Dorper rams subjected to artificial air conditioning was sensitive to the treatment carried out. Using principal component analysis, it was possible to identify the most important variables for characterizing seminal quality parameters, which enabled regression models to be formed, with the model generated for plasma membrane integrity being statistically strong and satisfactory.

In view of the findings, it is safe to say that the action of the adiabatic evaporative cooling system enhanced the natural changes that occurred in climatic variables during the study period, where an increase in relative humidity and a decrease in air temperature, within the animal’s comfort zone, were associated with positive benefits related to the plasma membrane integrity of the ram’s spermatozoa. Nevertheless, it should be noted that excessively high relative humidity values may reduce the efficiency of evaporative heat dissipation and, under certain conditions, predispose animals to respiratory challenges or pathogen proliferation.

Therefore, future studies should investigate the balance between temperature reduction and humidity increase to optimize the use of evaporative cooling systems in sheep production. In addition, long-term evaluations and studies involving other sheep breeds with different thermotolerance profiles are recommended to broaden the applicability of this environmental management strategy.

## Data Availability

The data is confidential.
